# Exploring COVID-19 pandemic side effects: The case of routine immunization in Afghanistan

**DOI:** 10.7189/jogh.12.03039

**Published:** 2022-07-22

**Authors:** Pedro Arcos González, Abid Zabibdullah, Jose Cernuda Martínez, Rafael Castro Delgado

**Affiliations:** Unit for Research in Emergency and Disaster, Department of Medicine, University of Oviedo, Oviedo, Spain

Immunization is a key component of primary health care systems and is globally accepted as a cost-effective intervention, preventing 2-3 million deaths annually, as well as illnesses and disabilities resulting from vaccine-preventable diseases [1]. The COVID-19 pandemic has seriously impacted individuals and communities across the globe, disrupted health systems, and significantly affected routine immunization (RI) [2]. To mitigate transmission and reduce impact, most countries started control measures that unfortunately disrupted their health systems. The United Nations International Children’s Emergency Fund (UNICEF), World Health Organization (WHO), and the Global Alliance for Vaccination and Immunization (GABI) warned that at least 80 million children under the age of one year are at risk of contracting vaccine-preventable diseases during the pandemic; the WHO even developed guiding principles of immunization services and priorities to strengthen and sustain primary health care including RI during the pandemic [3].

To face the COVID-19 pandemic, the Afghanistan government developed a National Emergency Response Plan for Coronavirus (NERPC) in March 2020 and implemented a lockdown in the last week of that month, closing academic institutions, public halls, markets, hotels, restaurants, and flights. During this period, shortages of health staff, medicines, routine vaccines, medical supplies, and diagnostic kits have seriously affected health facilities, limiting early diagnosis and treatment of COVID-19. Moreover, people were hesitant to seek health care services, including immunization. In March 2020, when the lockdown was instituted and polio immunization campaigns stopped due to COVID-19, the number of polio cases increased to 34 in Afghanistan, showing a reduction in immunization coverage [4]. During this time, health care access for the poor and vulnerable people was limited, further disrupting the country’s already fragile health system.

Our Research Unit conducted a preliminary research project to explore the impact of RI in the Langhman province of Afghanistan during the lockdown period (April-July 2020) compared to the same period in 2019. Primary health care services, including RI in Laghman province, were delivered through the execution of the World Bank-funded Basic Package of Health Services (BPHS) and Essential Package of Hospital Services (EPHS) in 67 different types of health facilities, 45 of which were providing RI. The program follows Afghanistan’s Expanded Program of Immunization (EPI) schedule through fixed and outreach services. Fixed services provide daily immunization inside heath facilities, whilst outreach services are held weekly or monthly in a non-health facility location, from which health workers can go out and return the same day. The aim of outreach services is to provide immunization to vulnerable individuals and communities with limited geographical access. EPI is one of the main subcomponents of the Basic Package of Health Services (BPHS) under the child health and immunization components in Afghanistan. This program functions under the Ministry of Public Health and provides 11 common vaccines cost-free to prevent vaccine-preventable diseases (VPD). These vaccines include oral polio vaccine (OPV), bacillus Calmette-Guerin (BCG), diphtheria-pertussis-tetanus (DPT), hepatitis B, Hemophilus influenza type B, measles, rotavirus vaccine, pneumococcal conjugative vaccine (PCV), and tetanus toxoid (TT).

**Figure:**
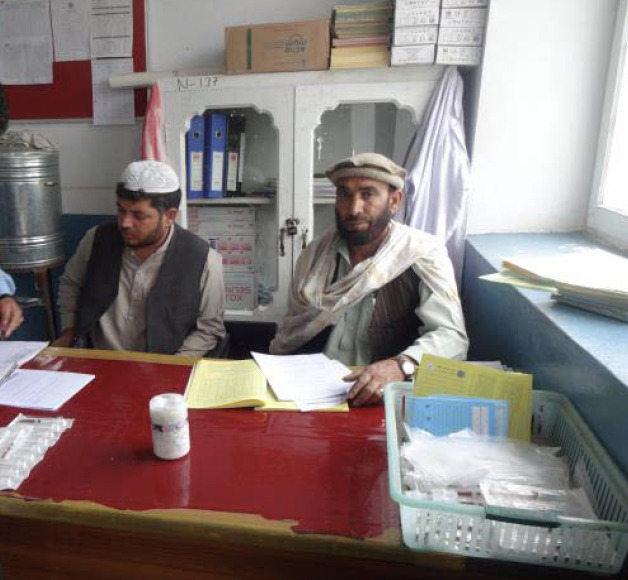
Photo: Members of the research project team at one of the Langhman province health centres. Source: From the authors’ own collection, used with permission.

We conducted a study to identify differences in vaccination coverage for girls and boys, fixed and outreach service availability, vaccination coverage between districts, and coverage by type of vaccination antigens. Four months of data were obtained from the Afghanistan EPI Health Management Information System database (EPI HMIS database). Data from two months during the lockdown (April-July 2020) and two comparison months (April-July 2019) were used. To obtain previously unrecorded data, a complementary study was conducted in 41 out of 45 BPHS health facilities providing RI services to the children in Laghman province. The preliminary findings show a decline of 21.4% in overall daily immunization coverage across all six districts of the province during the study period.

We found a reduction in immunization coverage across all vaccine antigens. The most affected antigens with a decline were measles (-28%) and OPV4 (-28%), followed by PCV3 (-26%), DPT3, IPV, OPV3, PCV2 and Rotavirus (-23%), DPT2 and OPV2 (-22%), PCV1 (-21%), Rota1 (-20%), BCG, Penta1, and OPV1(-19%), OPV0 (-18%) and Hep B (-6%). On average, a 56,1% decline was observed in outreach services and a 13% in fixed services. There was a 17% decline for boys and 26% for girls in total immunization coverage. The overall immunization reduction of 21.4% in Afghanistan was consistent with the study result found in England, where a 19.8% drop in Measles-Mumps-Rubella (MMR) vaccination was observed during the COVID-19 pandemic [5].

We also found a 56.1% decline in vaccine coverage through outreach and a 13.4% decline in fixed facilities. On average, 325 children per day missed out on lifesaving vaccination in Laghman province, putting them at risk of contracting vaccine-preventable diseases, especially polio and measles. Multiple factors might have caused this decline in immunization coverage, such as a reduction in health care provision, insecurity, disruption in global manufacturing and supply chains, border closure, and the global suspension by vaccine companies that led to shortages of vaccines and other supplies in the county.

Fear of COVID-19 exposure in health care settings also made people reluctant to leave homes and seek routine health care. A study in Saudi Arabia found that 60.9% of participants delayed getting their routine immunization due to fear of getting COVID-19 infection during the pandemic [6], supporting our findings. Additional reasons contributing to this delay, apart from lockdown measures and fear of transmission, were insecurity and frequent armed conflicts between the government and the opposition that limited access to health services.

A study in tertiary health care centres in India found a significant decline in attendance of routine immunization during the lockdown, mainly in May 2020 compared to April 2020, due to the pandemic peaking in the area [7]. Similarly, another study in Rajasthan India found a disruption to immunization services resulted in children missing immunization during the COVID-19 lockdown. This decline was largely found among children from poorer households, less educated and residing in the COVID-19 red zone [8]. In June 2020, a web-based survey conducted by WHO, UNICEF, and GAVI reported that out of 61 countries, 85% showed a low level of vaccination in May 2020 compared to January and February 2020, and 73% have reported a decrease in demand, mainly due to concerning COVID-19 infection, limited public transport, and maintaining physical distancing [9].

After the USA declared a national emergency, a reduction in routine immunization was observed, mostly in children older than one month [10]. An African study on COVID-19 and routine immunization suggested that risk of death due to vaccine-preventable disease outweighed the risk of death due to the possibility of contracting COVID-19 during clinic visits [11].

In our case, on average 325 children per day have missed out on their vaccination in the Afghan Laghman province across all antigens and all districts, risking exposure to vaccine-preventable diseases like measles and polio. There was also a high chance of secondary outbreaks during and after the pandemics. Locating these children and offering them vaccination can reduce the risks of VPD. It is important that the Afghanistan Ministry of Public Health, policymakers, and other stakeholders design specific strategies and interventions to sustain and strengthen primary health care services including RI, to increase vaccination coverage during pandemics and to prevent possible outbreaks of VPDs in the country.

Maintaining routine immunization during a pandemic following WHO guidelines, strengthening outreach and fixed services, ensuring supplies, capacity building of health workers, and massive campaigns have imperative roles in improving community awareness of the dangers of postponing vaccines and increasing the coverage during pandemics. To prepare and prevent future pandemics, we suggest that all countries implement tailored interventions and strategies to promote access and achieve a significant increase in the number of vaccinations during the pandemic, as well as to control the level of fear and anxiety provoked by the pandemics and media.
